# Climate change and the rising infectiousness of dengue

**DOI:** 10.1042/ETLS20180123

**Published:** 2019-04-16

**Authors:** Joacim Rocklöv, Yesim Tozan

**Affiliations:** 1 Department of Public Health and Clinical Medicine, Section of Sustainable Health, Umeå University, Umeå, Sweden; 2 College of Global Public Health, New York University, New York, NY, U.S.A.

**Keywords:** *Aedes*, arbovirus, climate, climate change, dengue

## Abstract

The disease burden of dengue has been steadily rising over the last half-century due to a multitude of factors, including global trade and travel, urbanization, population growth, and climate variability and change, that facilitate conductive conditions for the proliferation of dengue vectors and viruses. This review describes how climate, specifically temperature, affects the vectors’ ability to cause and sustain outbreaks, and how the infectiousness of dengue is influenced by climatic change. The review is focused on the core concepts and frameworks derived in the area of epidemiology of mosquito-borne diseases and outlines the sensitivity of vectorial capacity and vector-to-human transmission on climatic conditions. It further reviews studies linking mathematical or statistical models of disease transmission to scenarios of projected climate change and provides recommendations for future research directions.

## Introduction

Large and progressive reductions in the burden of many infectious diseases have been achieved in the past few decades through successful prevention and control efforts, improved sanitation and hygiene practices, and advances in medical care and treatment [1]. However, the global incidence of dengue has increased over the past three decades with large and periodic epidemics in endemic areas, outbreaks in immunologically naïve populations, and emergence in previously unexposed areas [2]. The reasons behind the unfettered global expansion of dengue are multifaceted; but the unprecedented increase in human mobility, both at the local and global scales, is likely to have driven it, along with rapid unplanned urbanization and climatic and environmental changes [3–8]. Equally important, this global expansion is set against a lack of safe and effective antiviral drugs and vaccines, despite significant increases in research and development funding for dengue over the same period [9].

The dengue virus belongs to the genus Flavivirus of the family Flaviviridae, and there are four genetically related but antigenically distinct serotypes of the virus [10]. After infection with a specific serotype, a person is thought to acquire lifelong immunity to that serotype [10]. A shorter period of cross-protection to other serotypes is also documented [11]. When the person gets re-infected with a second serotype, severe manifestations are likely to occur, including capillary leakage and hemorrhage (dengue hemorrhagic fever) and shock (dengue shock syndrome) [12]. Prompt diagnosis and adequate supportive therapy can decrease the case fatality rate in dengue patients, even among those with severe disease [13]. The annual symptomatic dengue infections are estimated to fall within the range of 50–100 million cases while dengue deaths at 10,000 per year [14], a significantly lower figure than the commonly cited estimate of 20,000 deaths per year by the WHO, which is most likely due to improved clinical management of patients, particularly with severe dengue [2].

Dengue is primarily spread by two species of *Aedes* mosquitoes: *Aedes aegypti* and *Aedes albopictus* [10]. Currently, *Ae. aegypti* is widespread in many tropical and subtropical regions of the world and is well adapted to urban environments [15], whereas *Ae. albopictus* has extended its range further to temperate areas and is more commonly found in rural and peri-urban environments [16]. Overall, both *Aedes* species are reported to be highly competent to transmit the dengue virus, but *Ae. aegypti* is considered to be the principal vector and is associated with large-scale epidemics globally [17]. The global expansion of dengue follows the global spread of its mosquito vectors. Climatic conditions, particularly temperature, constrain the geographical distribution and expansion of *Aedes* mosquitoes. Furthermore, temperature and precipitation strongly affect mosquito development and population dynamics. The average global temperature has been reported to increase by 0.8°C over the past century, at a rate of roughly 0.15–0.20°C per decade [18]. It is also widely projected that climate and weather variability will increase in a warmer world and exhibit a considerable spatial heterogeneity [19]. These projected changes in climatic conditions are predicted to affect the distribution and competence of these *Aedes* (and other mosquito) species as a vector and have a potentially significant impact on the epidemiology of dengue (and other vector-borne diseases) globally.

The purpose of this review is to synthesize the recent literature and describe the impact of climatic factors on dengue infectiousness and transmission dynamics with a specific focus on the primary dengue vectors. Key knowledge areas and future research areas are highlighted. We also review the complexity in estimating the impact of climate change on dengue dynamics given the dynamic interplay of social, biological, environmental, and economic factors that ultimately determine the risk of dengue infections in a population.

## Vector distribution

Currently, there is a lack of empirical surveillance of the dengue vectors, *Ae. aegypti* and *Ae. albopictus*, with sufficient global coverage to give a complete understanding of their presence, abundance, and seasonal activity. As a consequence, the presence/absence of these two vectors around the globe is predicted using interpolation methods based on sporadic observations of their known locations and climate, environmental, and socioeconomic variables across geographical areas. The most up-to-date maps using these methods show that these Aedes species are found in all continents, including North America and Europe [15]. Recent reports indicate a widespread distribution of *Ae. Albopictus* in Europe as a result of its dramatic spread in just the last couple of years [20]. Eggs and larvae of *Ae. Albopictus* have been found as far north in Europe as the U.K. [21], and the vector is predicted to expand even further across Europe [22], as well as in uncolonized areas of China [10].

## Vectorial capacity and basic reproduction number

Vector distribution and abundance are influenced by climatic conditions in many different ways in both the immature and mature stages of the lifecycle of mosquitoes. In the *immature stages*, vectors depend on fresh and clean water for breeding, preferably collected in small containers such as litter or used tires. Air and water temperature regulate the development of vectors from larvae to pupae. The two dengue vectors, however, differ markedly in their dependency on climate, and the most important distinction is the diapausing (hibernation) ability of *Ae. albopictus*, which is not observed in *Ae. aegypti*. This ability can be traced back to *Ae. albopictus* colonies in non-tropical Asia [23] and is now evident in this species in many subtropical and temperate areas of the globe [15]. Diapausing allows the vector to lay eggs that can survive the cold winter periods and hatch in the spring regulated by seasonal patterns of the length of days and temperature [24].

In the *adult stages*, vectors are affected by temperature predominantly for their survival and longevity. Temperature has a particularly important effect on the longevity of vectors and thus on their ability to survive long enough to replicate and transmit the dengue virus. The gonotrophic cycle describes the periods of blood feeding and connects the adult stage with the immature stage through oviposition, i.e. egg-laying. In a meta-analysis, the gonotrophic cycle at 30°C was estimated at ∼4 days for *Ae. aegypti*, and 4–6 days for *Ae. albopictus*, whereas it was at 20°C longer at ∼6 days and 7–15 days, respectively [3]. As the female vector feeds on blood for oviposition, the gonotrophic cycle is inversely related to the biting rate of the vector, which we denote by *a* in the vectorial capacity formulation below. A female mosquito can oviposit many times during lifetime as long as she survives long enough.

The period from a vector's exposure to the virus through a blood meal to when it is capable of transmitting it to a new host is described as the extrinsic incubation period, *n_m_*. The intrinsic incubation period and the survival rate of both Aedes species are temperature-dependent [4,25]. The vector has to outlive the extrinsic incubation period so as to transmit the virus to a susceptible human, which is less likely at low temperatures because the extrinsic incubation period is longer and the mortality rate is higher. The daily survival probability is denoted by *p*, and the instantaneous mortality rate is expressed as
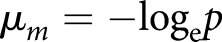
and hence *p* can be expressed as
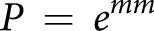
The relationship between vector survival and temperature has been studied extensively. *Ae. aegypti* has a high probability of survival for at least 40 days in experimental conditions when the temperature is between 20°C and 30°C, whereas *Ae. albopictus* has a similar high probability to survive in the same temperature range but for a longer period of 50–60 days [26]. At temperatures lower than 10°C, the longevity of both vectors is short, as is the case for *Ae. aedes* at 35°C and above and for *Ae. albopictus* at 40°C and above.

The ability of the vector to transmit the virus and the epidemic risk when the vector is exposed to the virus is considered in the vectorial capacity, which is also known as the daily reproduction rate. Originally, the vectorial capacity, *V*, was derived by Garret-Jones [27,28] and was expressed as
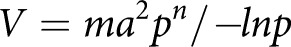
where *m* is the ratio of female vectors to human population. If the human population is stable, *m* can be estimated by the ratio of the emergence (hatching) rate of new adult mosquitoes to the mortality rate of adult mosquitoes, *µ_m_* [28]; *a* is the biting rate, *p* is the daily survival probability, and *n* is the extrinsic incubation period for the vector.

Virus replication in vectors is described by vector competence and is temperature dependent for ectotherm *Aedes* vectors [29]. Vector competence is estimated experimentally in healthy mosquito colonies by exposing them to the virus during blood feeding. If the virus is found in the abdominal guts of the vectors, it is considered to be infected. We describe this as a probabilistic event, denoted by *b_mi_*. if the virus spreads further and is found in the head and other peripheral parts of the vectors, it is considered to have successfully disseminated the virus. Furthermore, if the virus is found in the salivary glands and saliva of the vectors, it is considered to be capable of transmitting the virus. We also describe this as a probabilistic event, denoted by *b_mt_*. A vector is competent to transmit the virus only if the product of the probability of getting infected after blood feeding (*b_mi_*) and the probability of transmitting the virus through saliva (*b_mt_*) is larger than zero. The vector competence is computed by *b_m_* = *b_mi_b_mt_* and it is dependent on both average temperature and diurnal temperature variability [17].

Later, the vectorial capacity was also modified to include the vector competence for virus transmission (*b_m_*) and is now expressed as [30]:
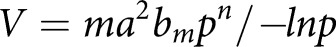
where the new term is defined as
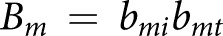
Vectorial capacity depends only on vector biology and is intrinsically related to the basic reproduction number for vector-borne diseases, *R*_0_, which is the expected number of hosts to be infected by a single infected host in a susceptible population [28] and is formulated as
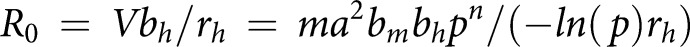
where *r_h_* is the recovery rate of infected humans (i.e. the infectious period), and *b_h_* is the probability of a susceptible host being infected if bitten by an infectious mosquito. *b_h_* has been estimated at 0.7 [4], and the average infectious period, *r_h_*, has been estimated at 5 days (range: 4–10 days) [25,31].

**Figure 1.**
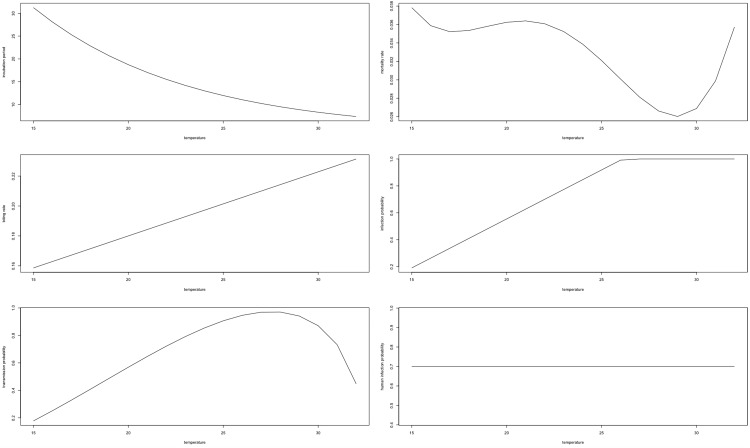
Temperature dependence of vectorial capacity parameters. Adopted from Liu-Helmersson et al. [5]: extrinsic incubation period (*n_m_*: top left), mortality rate (*µ_m_*: top right), biting rate (*a*: middle left), infection probability (*b_mi_*: middle right), transmission probability (*b_mt_*: bottom left), and probability of human infection (*b_h_*: bottom right).

Figure 1 presents the functional relationships between the parameters of importance for R_0_ and temperature for *Ae. aegypti*, as estimated by Liu-Helmersson et al. [5]. Note the change in denotations of *b_m_* = *b_mi_b_mt_*, which was referred to as *b_m_* and *b_h_* by Liu-Helmersson et al., and the additional parameter, *b_h_*, which was described above.

## Generation time of dengue transmission

The elapsed period of time from one cycle of vector-to-human transmission to the start of a new cycle is an important concept in the mathematical epidemiology of infectious agents and it is known as the generation time. The generation time for dengue transmission constitutes four elements: the extrinsic incubation period in vectors (*n_m_*), the time of vector-to-human transmission after the extrinsic incubation period, the intrinsic incubation period in humans (*n*_h_), and the time for human-to-vector transmission after the intrinsic incubation period. Considering the effect of temperature on the extrinsic incubation period in vectors (*n_m_*), the generation time highly depends on local climate and weather conditions. The other parameters are not known to be sensitive to temperature, but there is an almost linear negative relationship between the generation time and temperature, where we observe a generation time of 10 days at 35°C and above, 20 days at 30°C and above, and potentially a longer period of time at lower temperatures with considerable uncertainties [6]. Siraj et al. [6] used a simplified dynamic transmission model to estimate the growth rate of epidemics, which peaks at 33°C. In contrast, the parameters in the vectorial capacity, excluding the vector-to-human population ratio (m), are estimated to peak ∼28–29°C [4,5].

## Human-to-vector dengue transmission

**Figure 2.**
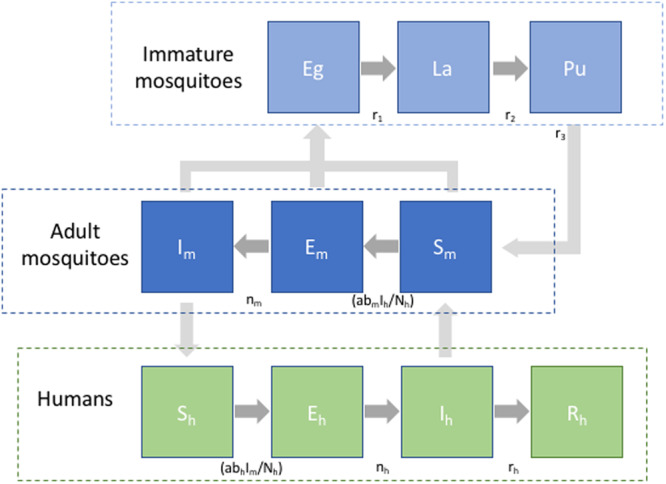
Graphical illustration of a compartmental transmission model with dynamic vector and human populations. Subscript *m* and *h* refer to mosquito and human, respectively, S meaning infected, E exposed, I infected, R recovered and Eg: egg, La: larvae, and Pu: pupae. Transition rates between the different compartments in the model are regulated by the rates indicated in between the compartments. Mortality and birth rates are not marked in the figure.

Human-to-vector dengue transmission systems are usually represented by compartmental process-based models that integrate feedback loops to describe the vector and human population dynamics and their interrelationships (Figure 2). Such models were developed first by Ross and then modified by Macdonald, and the theory has been recently described thoroughly by Smith et al. [28]. Using modeling frameworks, the vectorial capacity and the basic reproduction numbers can be solved numerically or solved analytically if less complex. In a simplified system, the immature stages are dependent on an environment conducive for female adult mosquitoes to survive, mate, blood feed, and oviposit her eggs (fecundity rate, *f*) in collections of clean water. The development from eggs to larvae and from larvae to pupae depends on nutrients available in breeding habitats, interventions targeting the immature stages of vectors, and water temperature, which will be close to ambient temperature for small containers. Climate-sensitive transition rates in the immature stages are denoted by *r*_1_–*r*_3_ in Figure 2. When pupae develop into adult mosquitoes and female mosquitoes start blood feeding, they can get infected by feeding on a viremic human. The likelihood of a mosquito feeding on an infectious human is estimated as the number of infected humans divided by the total human population, *I_h_/N_h_*. The mosquito will transition out of the susceptible state and enter the latent period of exposure depending on the probability of infection and transmission in the vector, *b_m_*, and will become infected depending on the temperature-regulated extrinsic incubation period, *n_m_*. After the extrinsic incubation period, adult mosquitoes can infect humans. The rate of susceptible to exposed and infected humans is derived as the product of the temperature-sensitive biting rate (a), the infection probability in humans (*b_h_*), and the ratio of infected mosquitoes to the total human population (*I_m_/N_h_*). The exposure to the infected rate is determined by the intrinsic incubation period, *n_h_*. All female mosquitoes blood feed and lay eggs. Infected, exposed, and susceptible mosquitoes are all assumed to experience the same mortality rate, *µ_m_*. In a more realistically representative transmission system, compartments can be divided into demographic stratums, exhibit growth and loss (birth and deaths rates), and include asymptomatic and symptomatic carriers and multiple dengue virus serotypes. In addition, migration in human populations can alter the rate of infected humans, imported infections, and exportation of infected humans, both in the short and long range. Additionally, the introduction of interventions to prevent or break transmission during outbreaks can modify the transition rates between the immature (larviciding and source reduction) or adult (fogging and insecticide spraying) stages, as well as the transition rates between the human stages of infection (antiviral drugs and vaccines).

A dynamic deterministic representation of the transmission dynamics is given below. The birth rate of the human population is given by the parameter *k*. Mortality rates are denoted by *µ*.

*Immature mosquito dynamics*
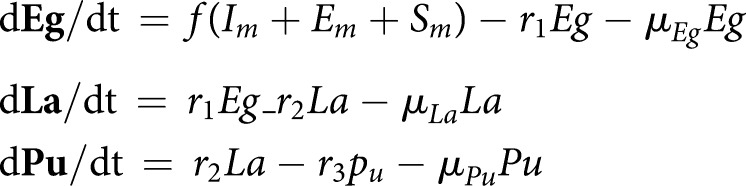
*Adult mosquito infection dynamics*
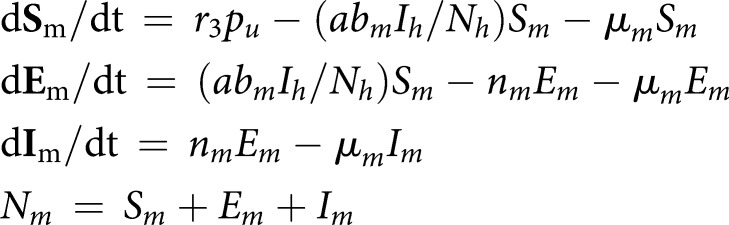
*Human infection dynamics*
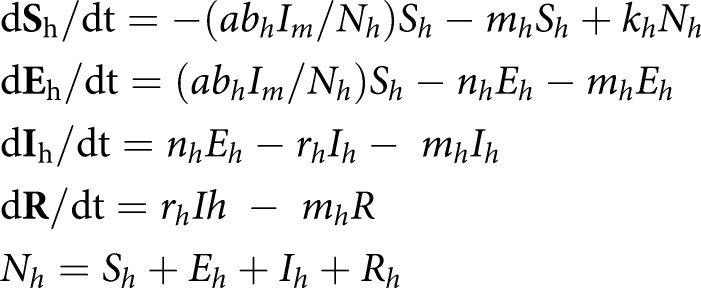


## Climate change impacts

A comprehensive analysis of the impacts of climate change on the incidence of dengue should consider the changes in a range of social, behavioral, economic, environmental, and health systems factors, including human mobility, vector control interventions, and potential vaccination coverage. These factors are important for determining the changes in exposure and susceptibility to the dengue virus in the face of climate variability and change. Because of the large uncertainties involved in future socioeconomic change scenarios, most studies have focused on assessing how the infectiousness of dengue and specific vector characteristics would change. The range and intensity of dengue transmission are assessed according to the climate projections described by the representative concentration pathways (RCPs). The RCPs correspond to a range of trajectories for greenhouse gas emissions and concentrations over the 21st century as described in the last IPCC report [32] and were developed alongside the global climate models (GCMs) in which the increases in greenhouse gas concentrations force changes in the climate conditions. The GCMs simulate the climatic changes regionally as well as globally and allow an assessment of climate change impacts across geographical and temporal scales [32]. Climate change impact analyses that use the GCM simulations and the RCP scenarios can assess the change in the biological hazard (i.e. the epidemic potential or the growth rate of transmission). Alternatively, the risk of disease can be assessed under the assumption of no demographic and socioeconomic changes. Generally, analyses examining climate change impacts use the scenarios of RCP2.6 to RCP8.5 (named according to the radiative forcing levels from 2.6 to 8.5 W/m^2^), corresponding to low- to high-emission futures [32]. Additionally, the inclusion of the socioeconomic development trajectories with population and economic changes, as described in the shared socioeconomic pathways (SSPs), can allow a more realistic assessment of disease incidence.

Using a simple model capturing temperature effects on vector development in the immature and mature stages, Siraj et al. found that the generation time of dengue transmission was highly sensitive to temperature, and the authors suggested that dengue epidemics may intensify as the temperature increases, because of not only higher numbers of infections per generation but also faster generation and hatching rates [6]. The optimal growth rate of epidemics at 33°C implied that many dengue endemic regions would be challenged by increased transmission potential in the future when temperature rises [6]. The authors made the observation that the average monthly temperature would exceed 33°C in India and African Sahelian regions in 2050 according to the climate change scenarios, and that the epidemic potential would reduce during the period from April to June in these regions. This analysis did not take the projected climate scenarios into account, and the analysis of climate change impacts was purely qualitative. It also did not consider vector competence, which has been shown to be sensitive to both low and high temperatures.

Liu-Helmersson et al. studied the change in the epidemic potential of *Ae. aegypti* globally using climate change scenarios from five GCMs that are downscaled and bias corrected to the 0.5–0.5 grid level (55 km by 55 km at the equator) [5]. This analysis projected the vectorial capacity by assuming a constant vector-to-human ratio (*m*) and investigated the effect of the changes in a diurnal temperature range in addition to the broader changes in the climate. Across different climate models, while a consistent increase was observed in the vectorial capacity in many sub-tropical and temperate areas, amplified due to the changes in diurnal temperature variability, the vectorial capacity was decreased in the hottest areas of the globe, including the Saharan desert, the African Sahelian region, and the Middle East [5]. The optimal epidemic temperature was estimated at 29.3°C when the diurnal temperature range was zero. However, at lower and higher temperatures, the vectorial capacity could reach high levels, if the diurnal temperature variation would allow parts of the days to be close to the optimal temperature [5]. This analysis differed from the analysis conducted by Siraj et al. [6] as it considered vector competence and diurnal temperature range and incorporated a simplified parameterization of the immature stages, which likely explain some of the differences in the findings of the two studies.

Liu-Helmersson et al. further studied the consequences of climate change on the vectorial capacity of the dengue vectors in Europe using a similar model but also included a simplified parameterization of the effect of temperature on the immature stages to approximate changes in the vector-to-human ratio (*m*) [4]. This analysis considered both *Aedes* vectors. The results showed that dengue would considerably extend its seasonal transmission window and geographical range of transmission. The analysis, however, showed that the vectorial capacity would increase to a level high enough to support epidemic growth with *R*_0_ greater than one in some areas, including parts of Italy, Greece, and Spain, if the dengue virus were to be introduced. The projections of the vectorial capacity of *Ae. aegypti* in Europe depend on the future expansion of this vector in mainland Europe. Liu-Helmersson et al. studied the risk of invasion of the vector using a dynamic process-based model and found the introduction in the continental Europe around the Mediterranean region to significantly depend on the degree of future warming, with a substantial higher invasion risk in Europe in the later part of the 21st century if carbon dioxide emissions follow the RCP8.5 trajectory [33]. The analyses by Liu-Helmersson et al. showed that *Ae. aegypti* could be already considered a competent vector for the dengue virus in Europe during summer and would become even more competent at higher temperatures, particularly under the high-emission scenario of RCP8.5 [4]. Liu-Helmersson et al. found a considerable difference in vector competence when contrasting the different RCPs to understand the climate-induced change in vectorial capacity. In particular, the vector competence of *Ae. aegypti* was considerably higher in the second half of the 21st century under the RCP8.5 emission scenario. Thus, Liu-Helmersson et al. concluded that mitigating greenhouse gas emissions could substantially reduce the risk of *Ae. aegypti* invasion and dengue transmission in continental Europe. Especially noteworthy is the recent upsurge of smaller arbovirus outbreaks in continental Europe caused by *Ae. albopictus* [34].

Lee et al. [35] simulated dengue incidence in Korea in future RCP scenarios using a model that incorporated immature and mature vector stages with temperature-dependent parameters and predicted an increasing incidence, assuming present-day conditions, and no developmental changes. Particularly, the contrast between RCP2.6 and RCP8.5 showed, similar to the study findings of Liu-Helmersson, to have a large impact on dengue incidence beyond the year 2050.

## Future research needs

Although great knowledge on the infectivity of dengue vectors has accumulated, many questions still remain to be researched and better understood. Chief among those is the relationship of vector competence to the different genomic strains of the dengue virus [36], which is the fitness of the virus to infect vectors. Studies have shown that vectors from different localities infected with the same virus strain appear to vary in their probability to develop infection, and that the genetic variability of vectors is important in this respect [36]. Therefore, it is important to investigate and describe these patterns in future research in order to better understand not only the variability but also in the epidemic potential of dengue and its geographical patterns. Moreover, further studies are needed to confirm the temperature dependence of the vectorial capacity parameters and their sensitivity to diurnal variability, developing further the research undertaken by Lambrechts et al. [17]. Such estimates are specifically needed for *Ae. albopictus*. Also, studies of other climatic determinants of vectorial capacity, such as ambient humidity, which has been shown to affect mosquito survival, would be of significant value. Furthermore, studies should better document and confirm the temperature-dependent vector competence of other arboviral diseases, including Zika, chikungunya, and yellow fever, that are likely to undergo changes similar to dengue in the future.

The studies should also seek to better estimate and include vector densities in the estimation of vectorial capacity and use vector density estimates that are field-validated. These estimates should be produced by taking into account not only the climatic factors, such as rainfall, temperature, and water temperature, but also land use and the capacity of a locality to suppress vector proliferation and the abundance of breeding habitats. Predictions for vector abundance under current and future climate have been inconsistent so far. An important caveat to bear in mind is that models incorporating few biological and environmental factors may lead to spurious results on vector abundance. Many of these factors are also likely to be affected by locality-specific socioeconomic and environmental conditions, as well as the effectiveness of vector control programs and interventions in place.

Studies of future impacts using social, environmental, and climate scenarios should seek to better use scenarios for land use, urbanization, population growth, human mobility, and microscale climate variability [37]. In relationship to temperature, studies that specifically incorporate urban heat islands are needed. In fact, urban heat islands can be several degrees higher than surrounding areas, and this can make a rather big difference to the survival and vectorial capacity of the vectors, particularly the urban dwelling *Ae. aegypti* vector, which can also be an important factor for infestation of this vector in fringe zones, such as the U.S.A., Europe, and China. We further emphasize the importance of understanding better the extent to which *Ae. aegypti* can adapt to human and artificial environments and how this species may circumvent the natural constraints of climate on its longevity, abundance, and virus replication.

Developing stochastic estimates of the risk of outbreak in non-endemic areas is another area where further research is needed, as the deterministic approaches are less appropriate for estimating outbreak risks and basic reproduction numbers when the incidence rate is low and subject to high stochasticity. The deterministic approaches are, however, sufficient and computationally effective for studying future risks in endemic situations, where the incidence rate is higher.

## Summary

- Arboviruses, particularly dengue, are on the rise globally. While dengue causes a large and increasing disease burden in endemic countries, recurrent outbreaks are observed outside the tropical zone.
- The vectors of dengue and other arboviruses, including chikungunya, Zika virus, and yellow fever, are intrinsically dependent on climate, specifically temperature, for their growth, survival, and feeding behavior, which regulate their potential to transmit these viruses. The epidemic potential can be described by vectorial capacity using compartmental models of disease transmission processes.
- The infectiousness of dengue is expected to increase further in many endemic and non-endemic regions according to the impact studies conducted using scenarios of projected climate change; however, these predictions are sensitive to the changes in future greenhouse gas emissions.
- Data on social, biological, environmental, and economic factors need to be incorporated into models to improve our ability to predict the actual transmission risk in an at-risk population in a dynamic climate.

## Abbreviations

GCMs: global climate models
RCPs: representative concentration pathways
SSPs: shared socioeconomic pathways
